# Exploring Microbiological Dynamics in a Salt Cavern for Potential Hydrogen Storage Use

**DOI:** 10.1111/1758-2229.70064

**Published:** 2025-03-12

**Authors:** Nicole Dopffel, Kyle Mayers, Abduljelil Kedir, Biwen Annie An‐Stepec, Janiche Beeder, Silvan Hoth

**Affiliations:** ^1^ NORCE Norwegian Research Center AS Bergen Norway; ^2^ Equinor ASA Stavanger Norway

**Keywords:** hydrogen underground storage, methanogenesis, salt caverns, sulphate reducing microbes

## Abstract

Hydrogen storage in salt caverns is important for supporting the energy transition. However, there is limited knowledge about microbial communities within these caverns and associated risks of hydrogen loss. In this study we characterised a salt‐saturated brine from a salt cavern and found a high sulphate content (4.2 g/L) and low carbon content (84.9 mg/L inorganic, 7.61 mg/L organic). The brine contained both Bacteria and Archaea, and 16S rRNA gene analysis revealed a halophilic community with members of *Acetohalobium*, *Thiohalorhabdus*, *Salinibacter* and up to 40% of unknown sequences. Within the Archaea, Euryarchaeota and the symbiotic Nanohaloarcheaota were dominant. Growth experiments showed that some microbes are resistant to autoclaving and pass through 0.22 μm filters. *Heyndrickxia*‐related colonies grew on aerobic plates up to 10% salt, indicating the presence of inactive spores. The highest anaerobic activity was observed at 30°C, including glucose‐ and yeast extract fermentation, hydrogen‐oxidation, lactate‐utilisation, methane‐ and acetate‐formation and sulphate‐reduction, which was observed up to 80°C. However, microbial activity was slow, with incubations taking up to 1 year to measure microbial products. This study indicates that artificial salt caverns are an extreme environment containing potential hydrogen‐consuming microbes.

## Graphical abstract

Hydrogen storage in subsurface salt caverns is vital for the energy transition. These caverns are not sterile, as they contain a diverse microbial world. Our research found Bacteria and Archaea thriving on hydrogen and other nutrients in this extreme environment.

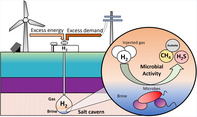

## 1 Introduction

To transition into a renewable energy system, energy storage is vital to buffer fluctuations in power production (IEA 2020) and power consumption. Wind and solar are dependent on local weather conditions and will not deliver a constant power load as fossil fuels. Because large amounts of surplus electricity from wind and solar cannot be stored in batteries, the energy must be converted into an energy carrier. Hydrogen (H_2_) has been proposed as an ideal solution (Chapman et al. 2019). H_2_ can be produced via electrolysis and then stored in large amounts. Afterwards, it can either be used directly as a feedstock or to generate power. To be able to store the volumes of H_2_ needed for a fully hydrocarbon‐independent energy system, geological storage or underground storage is the safest and most economical solution (Tarkowski 2019; Tarkowski and Uliasz‐Misiak 2022; Lemieux, Sharp, and Shkarupin 2019). Possible subsurface storage includes aquifers, depleted gas reservoirs, hard rock mines and salt caverns. H_2_ storage in salt caverns will likely be required in the near future because of the rather large cavern volumes (up to 500.000 m^3^), wide geological presence of suitable salt structures, low cushion gas requirements and very low H_2_ diffusion through the salt (Bérest and Louvet 2020; Caglayan et al. 2020; Laban 2020). Many of the existing caverns are currently used worldwide for gas, oil, brine and chemical storage, while only a few salt caverns are utilised for H_2_ storage, that is, Teeside UK since 1972 and along the US Gulfcoast since the 80's (Tarkowski 2019). Conversion of existing salt caverns to H_2_ use becomes increasingly important with growing H_2_ demand.

Despite their abundance, the microbial communities present in the salt caverns are not well understood. Salt caverns are not sterile and have conditions which are well within the microbial window of life when it comes to temperature (cavern temperature ranges from 20°C to 80°C) and cavern pressure (up to 300 bar). It is also known that salt caverns will always contain some level of brine at the bottom (sump) as a result of the leaching process (solution mining). This brine is typically saturated with salt, neutral in pH and very rich in sulphate, resulting from anhydrite dissolution. Few studies have shown halophilic communities present in different salt caverns around the world (Bordenave, Chatterjee, and Voordouw 2013; Schwab et al. 2022, 2023; Bock et al. 1994). The DNA community analysis of five different caverns in Germany (Schwab et al. 2022) showed different microbial communities in each cavern although some halophilic groups were present in several caverns. Due to variations in operational history, age, source of the leaching water and other factors unique to each salt cavern, it is possible that microbial communities within these caverns will exhibit significant differences, despite sharing some common physiochemical properties.

Microbial activity studies on salt cavern brines are very scarce, and the metabolic fluxes within salt caverns remain largely unknown. During H_2_ storage, these microbes will come in contact with high concentrations of H_2_, which is a ubiquitous electron donor supporting many different metabolisms. This could lead to activation of different H_2_‐consuming microbial groups including sulphate reduction, methanogenesis and acetogenesis resulting not only in H_2_ loss but also different microbial risks (Dopffel, Jansen, and Gerritse 2021; Gregory et al. 2019). This is especially true for sulphate reduction since sulphate as an electron acceptor can lead to significant losses of H_2_ and production of the toxic gas H_2_S. Methanogens and acetogens can use CO_2_ to produce either CH_4_ or the low‐molecular weight organic acid acetate. Schwab et al. (2023) showed that mixed cultures enriched from different caverns are able to consume H_2_ over time using both sulphate for sulphate reduction and also CO_2_ for acetogenesis. However, the occurrence, the rate and magnitude of these microbial processes within the salt caverns remains uncertain considering the energetically restraining high‐salt environment which can limit microbial activity (Oren 2011).

To gain more detailed insight into the microbial communities present in salt caverns and to better understand the metabolic diversity within, we sampled a salt cavern brine located in Northern Germany. This salt cavern has been leached within the Permian Zechstein Group. Using a variety of different methods, we first analysed the chemical composition of the brine and used both DNA‐based and culture‐dependent methods to gain an in‐depth understanding of the microbes present. The potential microbial activity, with a focus on relevant H_2_‐consumption processes, was assessed via enrichment studies at different temperatures over an incubation time of 1 year. Indeed, the salt cavern brine contains a diverse set of microorganisms including H_2_‐consuming halophiles with the potential of H_2_S formation. Understanding the magnitude of these metabolisms, their products and potential negative and positive feedback processes (Dopffel et al. 2023), should guide technologic and economic decisions related to H_2_ storage in salt caverns and hence support the energy transition. Our data provide insight into a largely unknown, human‐made and extreme environment.

## 2 Material and Methods

### 2.1 Sampling and Physical Parameters

Samples were taken in June 2022 in a salt cavern field located in Northern Germany. The cavern, which was leached in 2013, is located in a domal accumulation of the Permian Zechstein Group salt. This cavern was not used for hydrocarbon or any other product storage and hence continuously filled only with brine (volume ~ 190.000 m^3^). Brine samples were taken at the wellhead by pressure release. Before taking samples, the line was flushed for around 10 min and the first brine was discarded to avoid sampling the leftover brine standing in the well. The brine was filled into sterile and anoxic glass bottles (5 × 1 L) under continuous nitrogen flush to preserve anoxic conditions and then immediately shipped to our laboratory. Parameters like depth, pressure, temperature and cavern size were provided by the operator.

### 2.2 Chemical Analysis

pH was measured on an open membrane type pH‐meter (LAQUA twin pH‐22 compact, Horiba, Japan). Salinity (wt/wt) was measured with a standard pocket salt meter (ATAGO PAL‐1 refractometer, ATAGO co., LTD, Japan) against a 2.5% NaCl reference. Salinity (wt/v) was calculated from the measured density, which was determined by weight. Ionic composition was measured via inductively coupled plasma‐optical emission spectrometry (ICP‐OES) and ion exchange chromatography (IC). IC was also used to measure nitrate, sulphate and chloride. Alcohols, sugars and volatile fatty acids were analysed by using liquid chromatography on an Agilent 1260II UHPLC equipped with a flexible pump, autosampler, 1260 RI and 1260 DA HS detectors. All analytes were identified and quantified based on their respective reference standard calibration curves. The total and dissolved organic carbon and bound nitrogen were analysed by Elementar Vario TOC cube. Water activity was measured with Novasina LabMaster‐aw at 25°C.

### 2.3 DNA Based Analysis

For DNA analysis, 20 mL brine were filtered over a 0.22 μm filter directly on site. Three filters were prepared this way. They were immediately frozen over night at −20°C in a standard freezer located on‐site and then shipped frozen to the laboratory. Filtering higher volumes of brine, led to salt‐ and mineral precipitation on the filter, which inhibited DNA extraction (Sankaranarayanan et al. 2011). DNA was extracted from the filters using the DNeasy Power Soil Kit (Qiagen). DNA concentration was determined via Qubit dsDNA High Sensitivity assay (Invitrogen). To determine the copy numbers of different microbial groups, we used digital‐droplet PCR (ddPCR; BioRad) with standard 16S rRNA for Bacteria or Archaea (Ovreås et al. 1997), dsr1 primer for sulphate‐reducing bacteria (Kondo et al. 2004), mcrA primer for methanogens (Steinberg and Regan 2008) (Table S1). Copy numbers were calculated to cell numbers using assumed number of genes: 5.3 copies/cell for Bacteria and 1.7 copies/cell for Archaea (based on the rrnDatabase), 1 copy/cell for dsr1 and mcrA. Full PCR protocols, primer sequences, and targets can be found in Table S1. Since DNA concentrations were too low for amplicon 16S DNA sequencing to produce sufficient amounts of reads, DNA of two filters were randomly amplified using the Genomiphi V2 DNA Amplification Kit (Cytiva) according to manufacturer's instructions. Each sample was amplified in 10 separate reactions, and all products were combined to enhance statistical variation. (Kawai et al. 2014). Phi polymerase is suggested to be unspecific, but it can be assumed that the relative abundance will be also randomly affected. Doing the amplification 10 times in parallel, helps to get a more precise picture on the actual community abundance. However, introduced changes on the relative abundance might be still possible.

DNA was purified via bead purification prior to sequencing. The bacterial and archaeal community was obtained via Illumina Nextera two‐step libraries with the V3/V4 region of the 16S rRNA gene (341F/805R) (Connell et al. 2021) (R1: NNNNNCCTACGGGNGGCWGCAG; R2: NNNNNGACTACHVGGGTATCTAATCC) with 20 PCR cycles for the first step and 10 PCR cycles for the second step. Subsequently, the PCR libraries were sequenced on an Illumina MiSeq platform using a v2 500 cycles kit with 2*250 bp reads. Bioinformatic analysis included the following steps: produced paired‐end reads which passed Illumina's chastity filter were subject to de‐multiplexing and trimming of Illumina adaptor residuals using Illumina's bcl2fastq software version v2.20.0.422. The quality of the reads was checked with the software FastQC version 0.11.8. The locus specific V34 primers were trimmed from the sequencing reads with the software cutadapt v3.2. Forward and reverse reads were merged and quality filtered to in silico reform the sequenced amplicon using the software USEARCH version 11.0.667. The surviving reads were denoised using the UNOISE algorithm implemented in USEARCH to form amplicon sequence variants (ASVs) discarding singletons and chimeras in the process. Relative abundance tables were then filtered for possible barcode bleed‐in contaminations using the UNCROSS algorithm. ASV sequences were compared with the reference sequences of the NCBI RefSeq Targeted Loci database. Taxas were predicted and confidences were calculated using the SINTAX algorithm implemented in USEARCH. Alpha diversity calculations and the rarefaction analysis were performed with the R software packages phyloseq v1.30.0 and vegan v2.5–7. Library construction, sequencing and data analysis described in this section were performed by Microsynth AG (Switzerland).

Diversity index can be found in the Table S2. The whole amplification process was performed with ultra‐pure water. Analysis showed that the amplification process introduced background contamination, which could be identified by comparing the water controls to the actual samples. The ASVs attributed to the kit contamination were removed from the data set of the filter samples but can be found in Table S3. Taxonomic trees of the 16S reads were built using the online tool phyogeny.fr with one or two neighbours per ASV for Bacteria and Archaea (Dereeper et al. 2008, 2010; Edgar 2004; Castresana 2000; Guindon and Gascuel 2003; Anisimova and Gascuel 2006; Chevenet et al. 2006). All ASVs were deposited in ENA (European Nucleotide Archive under the project number PRJEB75363).

### 2.4 Microbial Cultivation and Enrichments

To screen for any aerobic non‐halophilic, and halotolerant aerobic species (which survive but are probably not active in the cavern), 100 μL of the brine was streaked on standard LB plates. The low salinity LB plate was chosen to remove the potential inhibitory effect sodium salts have on non‐halophilic microbial communities (Bonnet et al. 2020). Growing colonies were transferred on LB plates containing different amounts of NaCl ranging from 10% to 27% NaCl. Growth was followed visually. Incubation of the plates was at 30°C. For anaerobic cultivation, 25 mL of brine was filled in individual sterile bottles (total volume 58.35 mL) with different gas mixtures (100% H_2_ or 80%/20% H_2_/CO_2_ mixture or 100% N_2_) in the headspace. The bottles were closed with red butyl rubber stoppers. Different nutrient additions were used to investigate metabolic potentials: (a) 100% N_2_ + 10 mM glucose +0.2% yeast extract +0.2% peptone; (b) 100% N_2_ + 0.04% yeast extract, (c) 100% N_2_ + 20 mM lactate +20 mM acetate +0.04% yeast extract, (d) 100% H_2_ + 20 mM acetate and 0.04% yeast extract; (e) 80/20 H_2_/CO_2_ + 30 mM bicarbonate +10 mM formate, (f) 80/20 H_2_/CO_2_ + 30 mM bicarbonate +300 μL Postgate vitamin solution (g) 80/20 H_2_/CO_2_ + 30 mM bicarbonate, (h) 100% H_2_, (i) 100% N_2_. Set up (d) was designed to identify H_2_ consumers that might require additional minor nutrients for biofilm production and growth, trace yeast extract was added to supplement any potential micronutrients required. Set up (e) was designed to provide additional bicarbonate for microbial communities that require CO_2_ for their metabolism, that is, methanogen and acetogen. As the closest form of organic acid to H_2_, formate was supplemented. Set up (f) was designed similarly to set up (e) with the vitamin solution replacing the formate to investigate whether trace vitamin might boost the metabolic activity of cavern species and finally, incubation (g) was designed to provide bicarbonate and buffering of the incubation. A separate incubation with only H_2_ (set up h) was designed to screen H_2_ consumption without any additional boosters. Incubation temperatures were 30°C, 60°C and 80°C based on the information given by the operator at the time of sampling. Intended abiotic controls were: (a) autoclaved once and then either set up with 100% H_2_ or 100% N_2_ + glucose, yeast and peptone were added, (b) 100% N_2_ + filtration through 0.22 μm filter and glucose + peptone + yeast was added. All bottles containing H_2_ were stored upside down to minimise loss of H_2_ due to diffusion through the rubber stopper. Still, we observed diffusion through the stoppers, especially over longer incubation periods, which can lead to overestimation of H_2_ loss. This abiotic loss through stoppers was described before (Nauer et al. 2021) and is shown also in Figure S1. From active cultures, 5 mL culture were pelleted, and DNA was extracted and sequenced as described in the chapter before.

### 2.5 Sampling and Calculations

Pressure measurements, gas analysis and liquid sampling were performed in regular intervals during the incubation. Pressure in the bottles was measured using a pressure sensor from Sensortechnics 0–3 barg Press D/C 2916 with an individual set‐up for direct measurement of the headspace of serum bottles. Pressure in the headspace was measured at the start of sampling and at the end of sampling (when all gas and liquid samples have been withdrawn) to calculate the loss of H_2_ due to sampling. Gas composition was measured with a micro gas chromatography (microGC) 490 (Agilent) by directly measuring the gas in the headspace of the serum bottles. At some sampling points 1 mL of liquid was withdrawn for pH and HPLC determination. To calculate the amount of H_2_ in the bottles, the ideal gas law was used as described previously by (Dopffel et al. 2023). For each sampling point, the amount of H_2_ before and after sampling was calculated, to not overestimate H_2_ consumption. For determining the community of the active enrichments, we isolated DNA from 1 to 5 mL of sample, which was withdrawn using syringes and centrifuged for 20 min at 13,000 rpm. The pellet was frozen for several hours at −80°C and after that at −20°C for storage. This procedure was necessary because the pellets did not freeze at −20°C due to the high salt content. Prior to the DNA isolation step, the pellet was ultrasonicated for 5 min, frozen for several hours at −80°C, and again ultrasonicated 5 min. DNA was isolated using the DNeasy Blood & Tissue Kit (Qiagen) following manufacturer's instructions.

## 3 Results

### 3.1 Physical and Chemical Characteristics

Table 1 shows an overview of the salt cavern properties as well as the measured cavern brine chemistry. Sulphate content is over 4 g/L. Nitrate and iron were not detectable. Total organic and dissolved organic carbon are within the same range (~8 mg/L), so we assume that all organic carbon is present in the dissolved state. Sugars, alcohols or volatile fatty acids were not detected.

**TABLE 1:** Physical and chemical characteristics of the sampled cavern and cavern brine.

Physical cavern information	
Depth	1270–1525 m
Temperature	40°C–45°C
Pressure	34–43 bar
Volume	~190.000 m^3^

Chemical cavern information	
pH	7.48 ± 0.19
Salinity (wt/wt)	26.4% ± 0.5
Salinity (wt/v)	33.0%
Ionic strength	5.74 mol/L
Sulphate	4190.6 ± 57.5 mg/L
Nitrate	Below detection limit
Total inorganic carbon	84.9 ± 0.4 mg/L
CO_2_	0.3 ± 0 mg/L
H_2_CO_3_	0.24 ± 0 ug/L
${\mathrm{CO}}_{3}^{2-}$	35.3 ± 0.2 mg/L
${\mathrm{HCO}}_{3}^{-}$	49.4 ± 0.2 mg/L
Total organic carbon	7.61 ± 1.17 mg/L
Total bound nitrogen	0.61 ± 0.41 mg/L
Dissolved organic carbon	9.37 ± 0.45 mg/L
Dissolved bound nitrogen	0.60 ± 0.08 mg/L
Volatile organic compounds^a^	Below detection limit
Water activity	0.747 aw
Ionic composition	
Na^+^	124,587 mg/L
K^+^	457 mg/L
Ca^2+^	1022 mg/L
Mg^2+^	1032 mg/L
Cl^−^	198,471 mg/L
Fe	Below detection limit
Al	Below detection limit
Density	1.20 ± 0.00 g/mL

^a^ Includes sucrose, lactose, citric acid, glucose, fructose, pyruvic acid, succinic acid, lactic acid, glycerol, formic acid, acetic acid, methanol, propanoic acid, ethanol, buytric acid and butanol.

### 3.2 Microbiological Characterisation and Community

**FIGURE 1:**
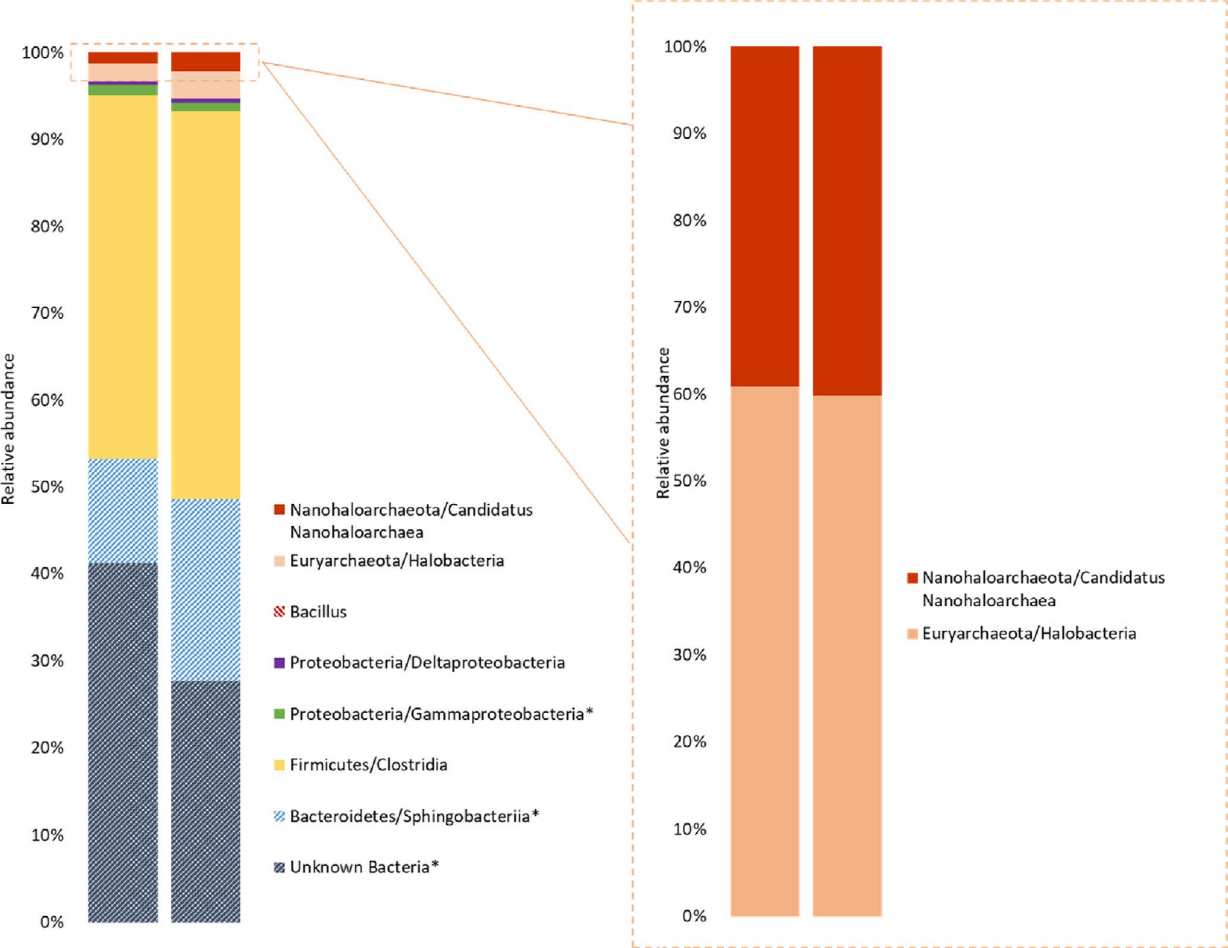
Relative abundance of identified major microbial taxa (left) and Archaea (right) according to 16S rRNA gene‐based amplicon sequencing of the original salt cavern brine. Two independent samples were amplified and sequenced and are shown separately. All ASVs are shown with the assigned taxonomy up to class level and confidence level of > 0.5. Taxa with * indicates a clustering of multiple ASVs with the same name. Corresponding ASV information are listed in Supporting Information.

**FIGURE 2:**
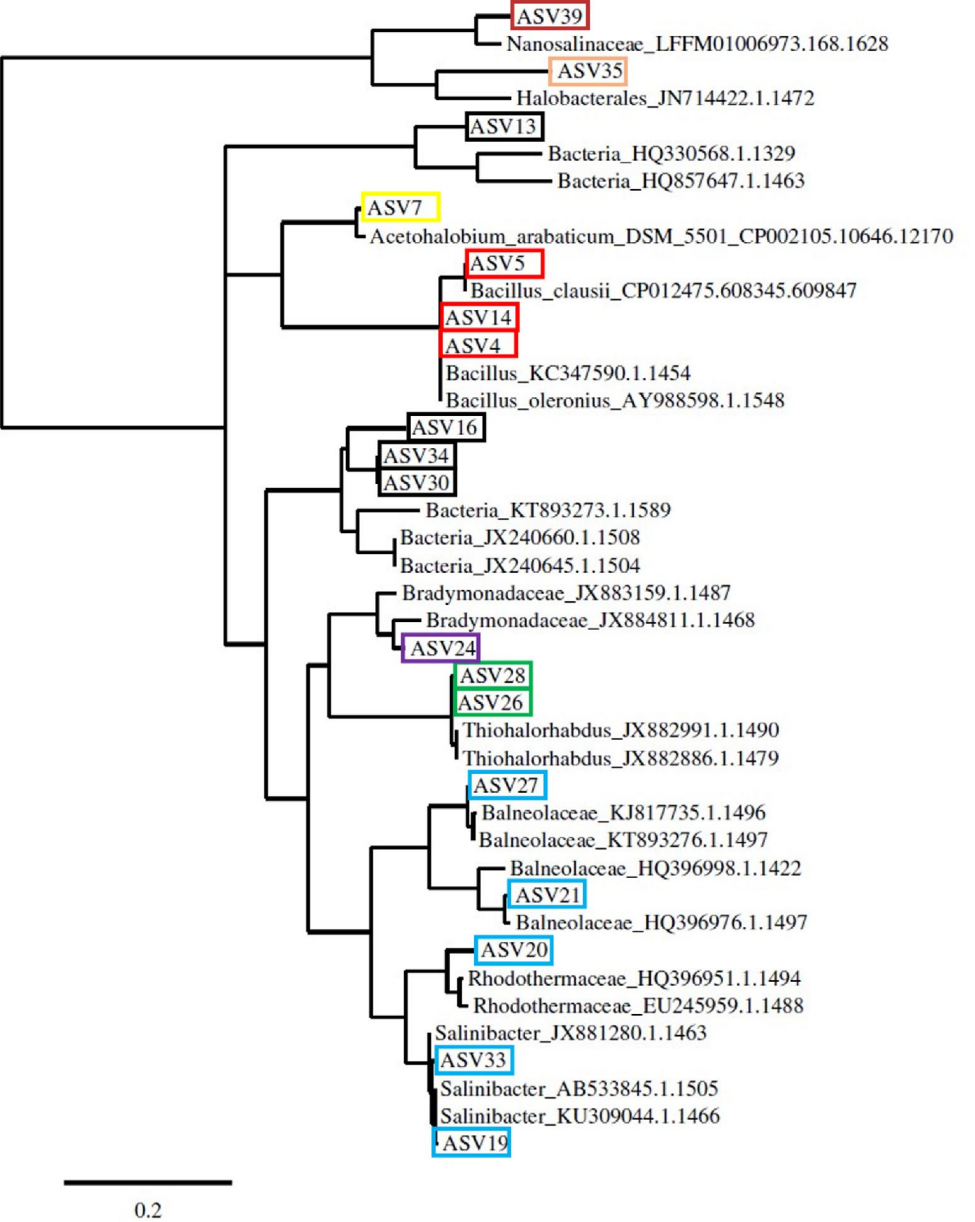
Phylogenetic tree based on the obtained 16S gene sequences. The bar represents the genetic distance. The closest neighbours (within RDP database) are used as reference. The ASVs are coloured corresponding to Figure 1 for better visibility. All ASVs are shown.

Bacterial and archaeal cell numbers were for Bacteria up to 3.6E+03 cells/mL and Archaea with up to 7.8E+04 cells/mL (see Table S4). Sulphate‐reducing bacteria could be detected of up to 6.8E+01 cells/mL but no methanogens were detected (S4). Overall, the DNA amounts which could be extracted were extremely low (0.07, 0.11, 0.16 ng/μL) (see Table S5). Filtering higher volumes of brine, we were not able to extract DNA, probably due to inhibition of the extraction chemistry. Extra freeze–thaw‐cycles and ultrasonication improved the DNA amounts only slightly. It was therefore required to amplify the DNA before sequencing as described in the method section (Yokouchi et al. 2006; Kawai et al. 2014) using the phi‐polymerase, and compared with an amplified water blank. Nevertheless, an amplification bias of the polymerase, which could change the relative community structure cannot be ruled out. ASVs contaminants visible in the water blank were removed from the data set before further analysis. The obtained relative abundance is shown in Figure 1 and Table S6 and the related phylogenetic tree is shown in Figure 2. Overall, 17 different ASVs were detected in the samples. The highest ASV abundance is related to Halobacteroidaceae/*Acetohalobium* (confidence 0.81). Other relevant members are Sphingobacteriales, some related to *Salinibacter* (confidence 0.96–0.99) or Balneolaceae (confidence 0.4), *Thiohalorhabus* (confidence 0.76–0.79), Deltaproteobacteria (confidence 0.6) (closet relative Bradymonadaceae) and a very small fraction (0%–0.06%) *Bacillus*. A large part of the community with 26%–38% is maximal identifiable at the domain level (Bacteria). Within the Archaea, we obtained two community members, Euryarchaeota (Halobacteria, confidence 0.59) and the symbiotic Nanohaloarcheaota (Nanosalina confidence 0.94).

### 3.3 Growth Characteristics

Aerobic growth was tested on standard LB plates incubated at 30°C. From 10 LB plates, nine showed colonies growing after some days up to 4 weeks. Four distinct colony shapes could be recognised with clear swarming behaviour. Slow growing colonies grow petal‐shaped (Figure S2). Colonies from each plate were pooled in one sample and sequenced. All colonies belonged to *Heyndrickxia* (formerly Bacillus), *Bacillus*, *Alkalihalobacillus*, *Micrococcus* and *Staphylococcus*. All identified ASVs per plate can be found in Table S7. Most ASVs are related to members formerly isolated from high salt, extremely dry or high temperature environments. Three ASVs are related to airborne microbes, which could indicate, that they are contaminations during sampling. Due to its unique colony shape, the colony shown in Figure S2A was further characterised. The strain is related to *Heyndrickxia oleronia* (formerly *Bacillus oleronia*), which is described to form highly resistant spores. The strain grows on LB up to 10% salt but not on LB with 27% salt, which is the cavern salinity. We therefore assume that the strain is in‐active as spores in the cavern.

Anaerobic growth results are shown as summary in Table 2. Overall highest activity was observed at 30°C. In some cases, only one of the duplicate bottles showed microbial activity. Fermentative growth on glucose and yeast extract + peptone triggered growth within 14 days at 30°C. Products of fermentation were CO_2_, H_2_, acetate and ethanol. When possible, we identified the community of the enrichments, which can be found in Table S8. The fermentative microbes were identified as *Halanaerobium* (confidence 1) and *Halanaerobacter* (confidence 0.99). We also observed some CO_2_ production at 60°C and 80°C and again the same halophilic members were detected via sequencing, at 80°C together with an unknown Bacteria (confidence 1). But no products could be detected in spent medium for 60°C or 80°C incubations. It therefore remains to be investigated, if the microbes can only shortly survive at 60°C and 80°C or if they can grow continuously at this temperature. The microbes are resistant to short‐term high temperature exposures is supported by the fact that the brine indeed showed again active fermentation at 30°C after autoclaving and furthermore, 0.22 μm filtration still showed fermentative growth (detected CO_2_ and H_2_ production over time via GC (Table S13 for autoclaved controls) or visually via increased turbidity for sterile filtered). The identity and more detailed research on these microbes are needed to further interpret these observations and the resulting conclusions for the community.

**TABLE 2:** Overview of enrichment cultures using the original salt cavern brine with different amendments incubated at different temperatures the after 340 days of incubation.

Growth conditions	*T* (°C)	H_2_S	pH increase	Acetate consumption	Black precipitates	CH_4_	Acetate production	Fermentation products
10 mM glucose, 0.2% yeast extract, 0.2% peptone								
	30	−	−	−	−	−	++	++
	60	−	−	−	−	−	−	−
	80	−	−	−	−	−	−	(+) traces of H_2_
Autoclaved brine + 10 mM glucose, 0.2% yeast extract, 0.2% peptone (single set‐ups)								
	30	−	−	−	−	−	nd	(+) traces of H_2_
	60	−	−	−	−	−	nd	−
	80	−	−	−	−	−	−	(+) traces of H_2_
0.04% yeast extract								
	30	++	−	−	++	+−	+−	−
	60	−	−	−	−	−	−	−
	80	−	−	−	+−	−	−	−
20 mM lactate, 20 mM acetate, 0.04% yeast extract								
	30	++	−	−	−	−	−	−
	60	−	−	−	−	−	−	++ (H_2_)
	80	−	−	−	−	−	−	
100% H_2_, 10 mM acetate, 0.01% yeast extract								
	30	++	++	++	++	+−	−	−
	60	−	−	−	++	−	−	−
	80	+−	−	−	+−	−	+−	−
H_2_/CO_2_, 10 mM formate								
	30	−	−	−	−	−	−	−
	60	−	−	−	−	−	−	−
	80	−	−	−	−	−	−	−
H_2_/CO_2_, vitamins								
	30	−	−	−	−	−	−	−
	60	−	−	−	−	−	−	−
	80	−	−	−	−	−	−	−
H_2_/CO_2_								
	30	−	−	−	−	−	−	−
	60	−	−	−	−	−	−	−
	80	−	−	−	−	−	−	−
H_2_								
	30	+−	++	−	++	−	−	−
	60	−	−	−	−	−	−	−
	80	−	−	−	−	−	−	−
Autoclaved brine + H_2_								
	30	−	++	−	−	−	−	−
	60	−	−	−	−	−	−	−
	80	−	−	−	−	−	−	−
N_2_								
	30	+ −	−	−	−	−	−	−
	60	−	−	−	−	−	−	−
	80	−	−	−	−	−	−	−

*Note:* + = clear sign of microbial activity like measurable gas or liquid products, (+) = minor signs of microbial activity like black minerals or traces of gas products, − = no signs of microbial activity. Results for all duplicates are shown (++ = activity in both bottles, +− = activity only in one bottle). n.d. = not determined.

Addition of only yeast extract showed H_2_S formation at 30°C after 94–136 days with values reaching 1082–2084 ppm in the headspace. Addition of lactate, acetate and yeast extract gave strong sulphate‐reducing activity at 30°C reaching over 10,000 ppm H_2_S in the headspace. We also observed H_2_S formation at 30°C with H_2_ + acetate + yeast (1350–1561 ppm), with H_2_ only (613 ppm) and traces with N_2_ only (37 ppm). With H_2_ + acetate + yeast, we observed the consumption of acetate, subsequent formation of CH_4_ (1372 ppm) and an increase in pH. Measured gas and pH values over time are given in Tables S9–S12. With only H_2_ in the headspace, we also observed a significant pH increase as reported earlier (Dopffel et al. 2023). We identified the community of the active bottles with lactate + acetate + yeast and H_2_ + acetate + yeast via 16S rRNA gene sequencing, which revealed a mixture of fermentative microbes including *Halanaerobium* and Halanaerobacter, probably growing on the yeast extract (Table S8). The H_2_S is produced by different ASVs from the sulphate‐reducing group Desulfohalobiaceae. Unknown Bacteria are present in both set‐ups. Nanohaloarchaeota is detected in the H_2_ set‐ups. Unexpectedly, we measured H_2_S (401 ppm) and acetate production (1.5 mM) at 80°C in the set‐ups with H_2_ + acetate + yeast. Up to this date, we were not able to identify the involved community, but we hope to further enrich the active microbes to get insight into the involved organisms. Some bottles contained black precipitates, which can be FeS resulting from H_2_S reacting with traces of iron. We detected no signs of activity in enrichments with CO_2_/bicarbonate added. However, the addition of CO_2_ led to a decrease in pH from 7.5 to 6.9 and resulted in mineral precipitations. Whether this is the reason for the lack of activity remains to be determined.

## 4 Discussion

### 4.1 Halophilic Community Present in the Salt Cavern

The chemical characteristics of the salt cavern brine are in line with other reports including a near neutral pH, very high sulphate‐ and low carbon content (Schwab et al. 2022). The sulphate probably stems from the dissolution of anhydrite (CaSO_4_) present within the Zechstein salt sequences (Cyran 2021). Salinity is around 33% (wt/v) which results in a water activity of 0.747 aw. We did not detect any volatile fatty acids or alcohols or sugar components. As the cavern was never in operation, there was no introduction of any workover fluids, so the original carbon from the sea water, as introduced during the leaching process, was probably used up relatively fast. What remains is a carbon‐ and nitrogen‐poor brine, which indicates nutrient limitations. Despite the low nutrients, the inferred cell numbers of both Archaea and Bacteria were relatively high indicating an active cavern community.

There are limited reports on the community present within salt caverns. Our investigated cavern fits to the general trend observed by Schwab et al. (2023), where two caverns were dominated by Archaea/Halobacteriaceae, one cavern was dominated by Halanaerobiaceae, another by Balneolaceae, with one cavern showing a very diverse microbiological community. We also detected both Archaea and Bacteria in our cavern, with the archaeal part of the community consisting of two members: Euryarchaeota/Halobacteria and Nanohaloarchaeota. The class Halobacteria was generally reported to be predominant in brine from saline lakes or salterns, and it was also detected at high abundance in some hypersaline sediments, such as soda‐saline sediment (Hartmann, Sickinger, and Oesterhelt 1980; Foti et al. 2007). They often grow aerobically with light, but some Halobacteria have the capability of strict or facultative anaerobic respiration based on elemental sulphur, dimethyl sulfoxide (DMSO) or nitrate. Halobacteria can also secrete low‐molecular‐weight organic acids and can grow chemoorganotrophic on amino acids. The other detected member belongs to Nanohaloarchaea of the DPANN (acronym of five candidate phylum names) superphylum. Taxonomic groups of this phylum were first detected less than a decade ago from Spanish saltern ponds and Australian salt lakes and seem to thrive in hypersaline ecosystems globally (La Cono et al. 2020). They are ectosymbiotic and rely on hosts, which can be, for example, Halobacteria. It was shown that Nanohaloarchaea can help the hosts to degrade more complex carbon sources like chitin and polysaccharides (La Cono et al. 2020). Based on the current literature data, all of them are aerotolerant, heterotrophic anaerobes relying on glycolysis, pyruvate fermentation, gluconeogenesis and glycogen synthesis/depolymerisation as their main energy pathways. They lack de‐novo biosynthesis of amino acids, purines and others. Genes for nitrite and sulphur reduction have been found but difficulties in the cultivation of DPANN taxonomic groups have prevented precise characterisation of their growth requirements (Zhao et al. 2022; La Cono et al. 2020).

The Bacteria community in our cavern was dominated with around 40% relative abundance by *Acetohalobium* belonging to Halobacteriodaceae. *Acetohalobium* has only one described member (*Acetohalobium arabaticum*) and it is growing on either chemolithoautotrophic H_2_ + CO_2_, methylotrophic growth using trimethylamine (TMA) or growing organotrophically using betaine, lactate, pyruvate or histidine (Sikorski et al. 2010). Other relevant members belong to *Thiohalorhabdus*, which are described as chemolithoautotrophic and facultatively anaerobic. They can oxidise inorganic sulphur compounds to sulphate or grow with thiosulfate or tetrathionate as electron donors and nitrate as electron acceptors. It is described that they are able to oxidise sulphide and elemental sulphur, but not H_2_ (Sorokin et al. 2008). Interestingly, according to the biogeographic pattern by Tu et al. (2022), abundance of both Thiohalorhabdaceae and Halobacteriodaceae are positively correlated with the total sulphur and inorganic carbon content of the environment, suggesting the association between sulphur‐containing minerals and carbonates in shaping the microbial community. Several ASVs are also related to *Salinibacter*, which are pigmented, extremely halophilic bacteria (Oren 2013). Up until now only aerobic growth on light and yeast extract is described although some genes related to anaerobic respiration on nitrite and sulphur compounds have been detected in the genome of *Salinibacter ruber*. Also, an amino acid metabolism is suspected to be encoded in the genome (Bagheri, Marashi, and Amoozegar 2019). As the artificial salt cavern is assumingly oxygen free and surely devoid of light, the present *Salinibacter* microbes clearly show that members of this genus can grow under dark and anaerobic conditions. A high amount of ASVs were only identified to the level Bacteria, which means that many unknown types of microbes are present in the cavern, which makes it difficult to predict microbial metabolisms. Testing of other primer regions for amplicon sequencing (Yang et al. 2022), especially other variable regions like V4‐V5, which cover the archaeal domain much better (Fadeev et al. 2021), full 16S sequencing or whole genome metagenomics would be helpful to obtain further information on the present microbes. However, such advanced methods are limited by the very low DNA yields and inhibition by high salt and other salt minerals, requiring further method optimization. The fact that DNA is very difficult to extract, some members of the community survive autoclaving and some seems to pass through 0.22 μm filters, indicates that the community members are small sized with very strong cell structures. This is interpreted as an adaptation to the high salt environment and combination of both osmotic and physical pressure, as a reduced cell size has been described to be a result of environmental stress (Kuhn et al. 2014). The combination of low brine volumes filtered, low extraction efficiency, PCR primer bias and cell passing through the 0.22 μm filters, and fact that we only observed low numbers of ASVs, indicates that we not fully captured the whole diversity of the cavern. To obtain the full range of the community, we recommend future investigations with optimised extraction protocols, additional primer regions and using a 0.1 μm filters as our current study may very well have missed more and important members.

In summary, the identified community structure suggests the presence of a diverse, yet largely uncharted, extremely halophilic community with the capacity for chemolithotrophic growth using both H_2_ and sulphur compounds, as well as potential involvement in amino acid‐ and osmotic solute cycling. This needs to be confirmed and further investigated in more extensive studies on both DNA and activity level. Fascinating is the fact that some ASVs relate strongly with ASVs reported by Belilla et al. (2019). They found similar halophilic ASVs in a saturated salt pond in a cave close to the Dallol area in Ethiopia. The deposited salt in this area is geologically not related to the salt found in Northern Germany, which opens the question how these specific extremophiles colonise these rare environments. More studies are needed to compare more salt cavern communities from different locations to understand how the microbial communities are shaped by the physical parameters.

### 4.2 Microbial Activity and Growth of the Halophiles Even at Very High Temperatures

Bacillus‐type ASVs were present in the original community sequences and were growing aerobically on low‐salt LB plates. Many ASVs relate to very dry, very alkaline or very saline environments and especially *Heyndrickxia* (formerly *Bacillus*) *oleronia* is described to produce extremely resistant spores (Fiedler et al. 2021). The role of these aerobic heterotrophs in the salt cavern is unclear but we assume they are not active but mainly present as spores. As Bacillus is a well‐known enzyme producer, the identified hyper salt‐tolerant Bacillus types might be a source of valuable new enzymes for biotechnology, which should be explored further (Outtrup and Jørgensen 2002). Interestingly, we did not see any sequences for *Halanaerobium* in the original filters but when sugar and other nutrients were added, members of the group were the dominant fermenting microbes. Halanaerobiaceae are very widespread halophiles, which have been found in fracking fluids and oil/gas reservoirs all over the world (Kögler et al. 2021; Booker et al. 2019). Although they seemingly do not play a major role in the nutrient‐deprived cavern, they do become dominant when complex nutrients are added. They were also activated by addition of only yeast extract. Overall, it seems that the addition of yeast extract did help to boost other microbial activity, since we saw much faster H_2_S production with added yeast (after 176 days) compared to without yeast (340 days). It could be that the yeast extract provides the limited nitrogen and phosphorus sources, or that the involved Halanaerobiaceae is providing certain amino acids or osmotic solutes to the remainder of the community. Complex interactions between halophiles have been described before (Booker et al. 2019) and we assume that this is also the case in this salt cavern. Overall, we have signs of active fermentation, sulphate reduction, methanogenesis and acetogenesis in the cavern brine at the tested enrichment temperatures. CH_4_ production was measurable only in bottles with yeast extract added where only traces of CO_2_ were present despite the fact that we did not detect related mcrA genes via PCR. The methanogens might be very low in cell number initially or we missed them in our DNA extraction approach. On the other hand, the fermenters could produce CO_2_ and H_2_ from the organics in the yeast extract, which then can be the source for both methanogens and acetogens.

The sulphate reducers in the cavern could be identified as members of the group of Desulfohalobiaceae, which includes the extremely halophilic members like *Desulfohalobium retbaense* (Ollivier et al. 1991), one of the most halophilic SRBs described up to date. Especially when lactate was added as an electron donor, H_2_S production was high at 30°C with over 1% in the headspace but it still took over 100 days before H_2_S was measured.

As a general trend, highest microbial activity was observed at 30°C. At 60°C, we only observed clear activity in the lactate enrichment. Surprisingly, we did observe activity (both sulphate reduction and seemingly acetogenesis) at 80°C in the H_2_ + acetate + yeast enrichment. Considering the salinity of 33% (wt/v), 80°C results in a significant stress on microbial life and there is no isolated strain reported to be able to grow under such conditions (Thaysen et al. 2021; Merino et al. 2019). This leads to a variety of fascinating questions about these hyperthermophilic, extremely halophilic microbes and how they can cope with the combined stresses of salinity and temperature. Alcaide et al. (2015) suggested that salinity might increase the temperature limit of enzymatic activity in deep sea environments. Considering that within a salt cavern there is the combined stresses of high salinity and pressure (around 40 bar, see Table 1), this could result in adaptations which also allow growth at high temperatures. We do not know yet which microbes are active in our enrichments and why they rather grow at 80°C than 60°C. Generally, with the exception of the sugar fermenting enrichment, activity was very slow, and it took months up to 1 year until any traces of significant activity were measured. However, we must acknowledge the fact that we did not have enrichments at the actual cavern temperature of around 40°C, as this info was not available at the time of the sampling. This means that activity might be more pronounced or faster or more diverse in the cavern compared to the 30°C enrichments. As the range 30°C–40°C is acceptable for many mesophiles, we can assume that the 30°C enrichments are the most representative for the actual cavern community.

### 4.3 Hydrogen Consumption and Implications for Storage

In an optimal case there will be no input of complex carbons into a salt cavern and only H_2_ will be injected. However, we do see microbial activity in bottles with only added H_2_. At 30°C, we observed H_2_S production and a concurrent pH increase, which we assume to be also relevant for the actual cavern temperature conditions. Based on studies from standard SRBs (Dopffel et al. 2023), the pH increase is linked to H_2_ and proton consumption, so we assume that the main part of the produced H_2_S comes from H_2_ oxidation. We see traces of H_2_S in the N_2_ enrichments also, which indicates that minor amounts can be produced without any addition of nutrients or electron donors. The sampled brine seems to be heavily nutrient limited, and activity is very slow. It can be assumed that the leaching water derived from the North Sea contained much more nutrients which were consumed over time. Addition of a complex nutrient source like yeast extract, gives insight into the potential of the microbial community present. Based on our results, it is anticipated that these processes will not occur to a significant degree in the cavern where nutrients are clearly limited, especially if nutrient input is avoided. Since, the brine contains a very limited amount of buffering components like carbonates, the triggered pH increase by sulphate reducers might lead to a self‐limiting feedback process, when pH optimum is exceeded (at least from the more abundant neutrophiles). It remains to be understood why we observe a very high abundance of *Acetohalobium* in the original sequences of the cavern, but we do only measure minor acetogenic activity. It is possible that these microbes fall within the ‘unculturable’ category, characterised by their inability to thrive in laboratory culture conditions, they grow at a different temperature, or they may exhibit significantly slower activity compared to sulphate reducers. Also, the necessary amplification of DNA prior to sequencing might have led to an artificial over‐representation of *Acetohalobium* sequences. Another option could be that produced acetate is directly consumed as carbon source by syntrophic microbes (especially the sulphate‐reducers) and therefore is not accumulating . Considering an incubation time of 1 year, it is clear that microbes in these extreme environments are active but at a different time scale compared with the duration of most microbiological research projects. Recently microorganisms have been identified from ancient halites from 800 million years ago (Schreder‐Gomes, Benison, and Bernau 2022). Whether such ancient microbes remain active or their very slow activity over years might be important for H_2_ storage in salt caverns, where the caverns are planned to be in operation for decades, needs to be further investigated. It could be that over years, the activity in the caverns will be noticed due to slowly increasing cell numbers or by the accidental sudden introduction of nutrients. If nutrient input is avoided, the expected H_2_ consumption activity seemingly does not pose a significant risk. Nevertheless, monitoring of the cavern during a H_2_ storage trials will be crucial to avoid unexpected activity. As the microbial cells are mostly located in the brine/sump phase, regular monitoring of the liquid phase will be the most insightful as H_2_S in the gas phase would already mean significant microbial activity. pH increase or increased cell numbers could also be monitored by capturing and analysing small aerosols formed during de/pressurisation of the cavern, which can be recovered and analysed during gas withdrawal operations (RD719132 2024; Bérest and Louvet 2020). Overall, microbial monitoring of salt caverns should be investigated and tested in future trials.

## 5 Conclusions

For safe and economically viable storage of H_2_ in salt caverns, the microbiological risks of H_2_ consumption and H_2_S release need to be assessed. The studied cavern harbours a diverse halophilic community with both Bacteria and Archaea including many unknown bacterial types. Activity tests showed a significant metabolic diversity and survivability of the community. Autoclaving and sterile filtration through 0.22 μm did not remove all microbes. Future studies on salt caverns should take these observations into account and adapt their methods accordingly (e.g., multiple rounds of autoclaving, using smaller filters). The presence of salt tolerant, heterotrophic spore formers also indicate that many microbes endure in the cavern although not being active. The brine itself is strongly nutrient limited and addition of yeast extract boosted the microbial activity when growing in the presence of H_2_. Even without yeast extract, H_2_ consumption and H_2_S formation were detected. However, both reactions were very slow, and incubations took up to 1 year to measure any significant microbial products. Further studies and field trials are needed to fully capture the diversity of this extreme habitat, using optimised DNA sampling and sequencing methods and more in‐depth growth tests at more temperatures. Our study provided the first baseline of the presence and potential activity of microbes in artificial salt caverns, which are a fascinating extreme environment with the potential discovery of polyextremophilic microbes. We indeed can see microbial activity at 80°C, which opens new questions about microbial survival strategies.

## Author Contributions

**Nicole Dopffel:** conceptualization, methodology, writing – original draft. **Kyle Mayers:** methodology, writing – review and editing. **Abduljelil Kedir:** methodology. **Biwen Annie An‐Stepec:** writing – review and editing. **Janiche Beeder:** conceptualization, writing – review and editing. **Silvan Hoth:** conceptualization, writing – review and editing, resources.

## Conflicts of Interest

Silvan Hoth and Janiche Beeder are employed by Equinor, which is researching hydrogen storage in salt caverns. The other authors declare no conflicts of interest.

## Supporting information


**Data S1.** Supporting Information.

## Data Availability

All main data generated or analysed during this study are included in this published article (and its Supporting Information files). Sequences are available online.
