# Supplementary material for: Exploring Microbiological Dynamics in a Salt Cavern for Potential Hydrogen Storage Use
Source: Environ Microbiol Rep. 2025 Mar 12;17(2):e70064. doi: 10.1111/1758-2229.70064 (PMC11903319; doi:10.1111/1758-2229.70064)
Supplement: Supplementary file 1 — Data S1. Supporting Information. [file EMI4-17-e70064-s001.docx]

Exploring microbiological dynamics in a salt cavern with potential use for hydrogen storage

SUPPLEMENTAL INFORMATION

Nicole Dopffel^1*^, Kyle Mayers^1^, Abduljelil Kedir^1^, Biwen Annie An-Stepec^1^, Janiche Beeder^2^, Silvan Hoth^2^

^1^ NORCE Norwegian Research Center AS, Norway

^2^ Equinor ASA, Norway

*To whom correspondence should be sent:

Nicole Dopffel, - NORCE

Nygårdsgaten 112, 5008 Bergen, Norway

Phone: +47 56 10 71 51

Email: nicd@norceresearch.no

**Table S1: Full PCR protocols, primer sequences, and targets**

| **Final primer sets Target** | **Forward** | **Reverse** | **Temperature** | **Concentration** | **Other** |
| --- | --- | --- | --- | --- | --- |
| Bacteria | L/Prba338f | K/Prun518r | 61 | 250 nM | Standard ddPCR protocol* |
| Archaea | Arch93f | mPrea1100r | 63.1 | 250 nM | Standard ddPCR protocol* |
| Sulphate reducers | dsr1F+ | dsrR | 57.1 | 250 nM | Standard ddPCR protocol* |
| Methanogens | mlas | mcrA-rev | 55 | 250 nM | Protocol adjusted; 45x cycles, annealing time 2 min, denaturation time 1 min and ramp rate of 1°C/sec |

* Standard ddPCR protocol is 95°C for 15 minutes and then 40 cycles of 95°C for 30s (denaturation) and annealing temperature (see table) for 1 minute followed with 4°C for 5 minutes, 90°C for 10 minutes.

The ddPCR reactions were run with a total volume of 20 µL on a DX200 instrument (BioRad) using 1x EvaGreen supermix (BioRad) and 250 nM (final concentration) of primers. Complete PCR reactions were emulsified with QX200 Droplet Generation Oil for EvaGreen using the QX200 Droplet Generator and then transferred to a 96-well plate. PCR reactions were performed in a C1000 Touch Thermocycler with deep-well module (BioRad) using the following program: 95°C for 15 minutes, 40 cycles of 95°C for 30 seconds, 57.1°C (sulphate-reducing bacteria) or 63.1°C (archaea) for 1 minute, 4°C for 5 minutes, 90°C for 10 minutes and finally an infinite hold at 4°C. Plates were equilibrated to room temperature for at least 10 minutes before being analysed on a QX200 Droplet Reader (BioRad). Thresholds for positive and negative droplets were manually set using positive and negative (ultra-pure water) controls.

**Table S2: Observed ASV and observed diversity index**

|  | **Sample** | **Reads** | **Observed ASVs** | **Simpson Index** | **Shannon Index** |
| --- | --- | --- | --- | --- | --- |
| Filter_original_phi | Sample 05 | 20452 | 35 | 0.219 | 2.05 |
| Filter_original_phi | Sample 07 | 13152 | 33 | 0.232 | 2.03 |
| CONTROL_phi | Sample 13 | 5396 | 21 | 0.0654 | 2.85 |

**Table S3: Phi polymerase kit contamination ASV identity**

| 1 | Proteobacteria | Betaproteobacteria | Hydrogenophilales | Hydrogenophilaceae | Tepidiphilus | Tepidiphilus_margaritifer |
| --- | --- | --- | --- | --- | --- | --- |
| 2 | Proteobacteria | Alphaproteobacteria | Sphingomonadales | Sphingomonadaceae | Sphingomonas | Sphingomonas_ginsenosidimutans |
| 3 | Actinobacteria | Actinobacteria | Actinomycetales | Micrococcaceae | Arthrobacter | Arthrobacter_russicus |
| 4 | Proteobacteria | Gammaproteobacteria | Pseudomonadales | Pseudomonadaceae | Pseudomonas | |
| 5 | Proteobacteria | Alphaproteobacteria | Rhizobiales | Methylobacteriaceae | Methylobacterium | |
| 6 | Proteobacteria | Betaproteobacteria | Burkholderiales | Comamonadaceae | Pelomonas | |
| 7 | Proteobacteria | Gammaproteobacteria | Enterobacteriales | Enterobacteriaceae | EscherichiaShigella | |
| 8 | Firmicutes | Bacilli | Lactobacillales | Enterococcaceae | Enterococcus | Enterococcus_faecalis |
| 9 | Proteobacteria | Gammaproteobacteria | Enterobacteriales | Enterobacteriaceae | Salmonella | |
| 10 | Tenericutes | |  |  |  |  |
| 11 | CyanobacteriaChloroplast | Chloroplast | | Chloroplast | Bacillariophyta | |
| 12 | Firmicutes | Bacilli | Bacillales | Staphylococcaceae | Staphylococcus | |
| 13 | Proteobacteria | Alphaproteobacteria | Rhizobiales | Bradyrhizobiaceae | Bradyrhizobium | |
| 14 | Proteobacteria | Alphaproteobacteria | |  |  |  |
| 15 | Firmicutes | Bacilli | Bacillales | Bacillaceae_1 | Bacillus | Bacillus_mojavensis |
| 16 | Proteobacteria | Alphaproteobacteria | Rhizobiales | |  |  |
| 17 | Firmicutes | Bacilli | Bacillales | Listeriaceae | Listeria |  |
| 18 | Firmicutes | Bacilli | Lactobacillales | Lactobacillaceae | Lactobacillus | Lactobacillus_fermentum |
| 19 | Proteobacteria | Alphaproteobacteria | Rhizobiales | |  |  |
| 20 | Thaumarchaeota | | Nitrososphaerales | Nitrososphaeraceae | Nitrososphaera | |
| 21 | Acidobacteria | Acidobacteria_Gp6 | |  | Gp6 |  |

**Figure S1: Control experiment of bottles containing 100% hydrogen and distilled water only. The bottles were closed with red butyl rubber stoppers and incubated at 30°, 60° and 80°C. Loss of hydrogen in % is shown. The data shows that the hydrogen is leaking out of the bottles via the stoppers. Leaking increases with increasing temperature.**

**Table S4: Cell numbers per mL brine determined via ddPCR of the different microbial groups. Results for all 3 filters are given.**

| **Group** | **Cells/mL** |
| --- | --- |
| Total Bacteria (16S) | Filter 1: 1.5 E+03  Filter 2: 2.3 E+03  Filter 3: 3.6 E+03 |
| Total Archaea (16S) | Filter 1: 2.7 E+04  Filter 2: 4.1 E+04  Filter 3: 8.6 E+04 |
| Sulphate-reducing bacteria (dsr1) | Filter 1: 1.1 E+01  Filter 2: 7.0 E+00  Filter 3: 6.8 E+01 |
| Methanogenic archaea (mcrA) | Below detection limit (<1.7 cells/mL) |

**Table S5: DNA concentrations and quality control**

|  | **DNA QuBit (ng/ul)** | **DNA nanodrop** | **A260/280** | **A260/A230** |
| --- | --- | --- | --- | --- |
| **Filter 1** | 0.07 | <0 | 5.02 | 0.02 |
| **Filter 2** | 0.111 | 5.7 | 1.47 | 0.03 |
| **Filter 3** | 0.165 | 2 | 0.87 | -0.02 |

**Table S6: Identified Bacteria of the original salt cavern brine. For RDP confidence value of 0.5 (=50%) are used. In the NCBI BLASTn (https://blast.ncbi.nlm.nih.gov/Blast.cgi) the highest confidence result is shown.**

| **ASV** | **RDP database (confidence until 0.5)** | **SILVA database (confidence until 0.5)** | **NCBI BLASTn** | **Information** |
| --- | --- | --- | --- | --- |
| 13 | Bacteria (0.99) | Patescibacteria; ABY1; Candidatus Magasanikbacteria; | Uncultured bacterium clone Dallol_cave_7GT (0.88) | Saturated salt pond in a cave in the Dallol area in Ethiopia |
| 16 | Bacteria (1) | Bacteria | Uncultured bacterium clone Dallol_cave_7GT (1) | Saturated salt pond in a cave in the Dallol area in Ethiopia |
| 30 | Bacteria (1) | Firmicutes | Uncultured bacterium clone OM (0.99) | Hypersaline Cyanobacterial Mats Oman |
| 34 | Bacteria (1) | Firmicutes | Uncultured bacterium clone OM (0.99) | Hypersaline Cyanobacterial Mats Oman |
| 19 | Salinibacter ruber (0.8352) | Salinibacter | Uncultured bacterium clone Dallol_cave_7GT (1)  OR  Salinibacter altiplanensis strain AN15 (0.957) | Saturated salt pond in a cave in the Dallol area in Ethiopia  Aerobic from hypersaline lake in Argentina, pigmented. Can use sugars and some amino acids, Anaerobic growth with arginine or DMSO |
| 20 | Sphingobacteriales (0.5514) | Rhodothermaceae | Uncultured bacterium clone OM_int_bact320 (1)  OR  Rhodothermus profundi strain DSM 22212 (0.882) | Hypersaline Cyanobacterial Mats Oman  Aerobic, Thermophilic strain from deep sea hydrothermal vent. Can use different amino acids and organic acids |
| 21 | Sphingobacteriales (0.7683) | Balneolaceae | Uncultured Bacteroidetes bacterium clone HAHS13.018 (0.98)  OR  Fodinibius salinus strain DSM 21935 (0.87) | haloalkaline soil India  halophilic from salt mine China, can reduce nitrate, pigmented |
| 27 | Sphingobacteriales (1.0000) | Balneolaceae | Uncultured bacterium clone xjeb-bact93 (1)  OR  Aliifodinibius saliphilus strain ECH52 7 (0.87) | sediment of Ebinur salt lake China  Aerobic, pigmented, sugars used, halophilic |
| 33 | Salinibacter_ruber (0.9702) | Salinibacter | Uncultured bacterium clone Dallol_cave_PGT_B1  OR  Salinibacter ruber strain P18 (0.95) | Saturated salt pond in a cave in the Dallol area in Ethiopia  Extreme halophilic, from crystallizer ponds, grows on amino acids, pigmented |
| 7 | Acetohalobium (0.8100) | Halobacteroidaceae | Uncultured bacterium clone BacB5-LacFum (1)  OR  Acetohalobium arabaticum DSM 5501 (0.98) | Hypersaline Sediment  Halophil, Strict anaerobic, grows on CO, CO2, H2, TMA, betaine, lactate, pyruvate |
| 26 | Thiohalorhabdus_denitrificans (0.6585) | Thiohalorhabdus | Uncultured bacterium clone Dallol_cave_7GT_B1_21 (1)  OR  Nitrosococcus watsonii C-113 (0.87) | Saturated salt pond in a cave in the Dallol area in Ethiopia  Aerobic ammonia oxidizer |
| 28 | Thiohalorhabdus_denitrificans (0.7050) | Thiohalorhabdus | Uncultured bacterium clone Dallol_cave_7GT_B1_21 (0.997)  OR  Nitrosococcus watsonii C-113 (0.87) | Saturated salt pond in a cave in the Dallol area in Ethiopia  Aerobic ammonia oxidizer |
| 24 | Deltaproteobacteria(0.6300) | Bradymonadaceae | Uncultured bacterium clone OM_int_bact098 (0.99)  OR  Persicimonas caeni strain YN101 (0.88) | Hypersaline Cyanobacterial Mats Oman  From solar saltern China, moderate halophile, pigmented, grows on sugars, some amino acids |
| 14 | Bacillus oleronius (0.88) | Bacillus | Heyndrickxia oleronia J19TS1 (0.997) | High heat tolerance, from Dairy products |
| 4 | Bacillus oleronius (0.9000) | Bacillus | Heyndrickxia oleronia J19TS1 (1) | High heat tolerance, from Dairy products |
| 5 | Bacillus (1) | Bacillus | Alkalihalobacillus shacheensis HNA-14 (0.97) | High salt and alkali tolerance, from rhizosphere |
| 35 | Euryarchaeota (0.9118)/ Halobacteria (0.5927) | Halobacteriales | Uncultured archaeon clone Dallol_cave_GT (0.95) | Saturated salt pond in a cave in the Dallol area in Ethiopia |
| 39 | Nanohaloarchaeota (0.9700)/ Candidatus_Nanosalina (0.9409) | Candidatus Nanosalina | Uncultured Nanohaloarchaeota archaeon clone N_TG86 (0.989) | Hypersaline Aquatic Habitats, Turkey |

**Table S7: Identified microbes from original salt cavern brine streaked on LB plates. For RDP only confidence value of 0.8 (=80%) are used. In the NCBI microbe BLAST the highest confidence result was used.**

| Sample | Growth detected? | | RDP identity (confidence in relative %) | NCBI BLAST Microbes (confidence) | | Information |
| --- | --- | --- | --- | --- | --- | --- |
| Bottle 1 | Y | | *Micrococcus (0.99)*  *Micrococcus (0.98)*  *Bacillus oleronius (0.88)*  *Bacillus oleronius (0.8429)*  *Bacillus (0.9409)*  *Bacillus oleronius(0.9000)* | *Micrococcus aloeverae M71 (1)*  *Micrococcus luteus CW (1)*  *Heyndrickxia oleronia J19TS1 (0.997)*  *Heyndrickxia oleronia J19TS1 (0.997)*  *Bacillus haynesii P19 (1)*  *Heyndrickxia oleronia J19TS1 (1)* | | High salt tolerance, from Aloe Vera  Air borne in school  High heat tolerance, from Dairy products  High heat tolerance, from Dairy products  High salt/alkali tolerance, from tree  High heat tolerance, from Dairy products |
| Bottle 1 | | N | | | *-* | |
| Bottle 2 | Y | | *Micrococcus (0.99)*  *Micrococcus (0.98)* | *Micrococcus aloeverae M71 (1)*  *Micrococcus luteus CW (1)* | | High salt tolerance, from Aloe Vera  Air borne in school |
| Bottle 2 | | N | | | *-* | |
| Bottle 3 | Y | | *Bacillus (0.9409)*  *Bacillus (0.9025)*  *Bacillus(0.9409)* | *Bacillus haynesii P19 (1)* | | High salt/alkali tolerance, from tree |
| Bottle 3 | Y | | *Bacillus (0.9409)*  *Bacillus (0.9025)*  *Bacillus(0.9409)* | *Bacillus haynesii P19 (1)*  *Bacillus licheniformis sMT-B06 (1)*  *Bacillus licheniformis MMI12C (1)* | | High salt/alkali tolerance, from tree  From Fermented wheat  From Fermented wheat |
| Bottle 4 | Y | | *Micrococcus (0.99)* | *Micrococcus aloeverae M71 (1)* | | High salt tolerance, from Aloe Vera |
| Bottle 4 | Y | | *Bacillus (1)*  *Bacillus (1)*  *Bacillus (1)* | *Alkalihalobacillus patagoniensis (0.97)*  *Alkalihalobacillus shacheensis HNA-14 (0.97)*  *Alkalihalobacillus patagoniensis (0.97)* | | High salt and alkali tolerance, from rhizosphere  High salt/alaki tolerance, from desert soil  High salt and alkali tolerance, from rhizosphere |
| Bottle 5 | Y | | *Staphylococcus (1.0000)*  *Staphylococcus (1.0000)*  *Staphylococcus (1.0000)* | *Staphylococcus caeli strain 82B (0.99)*  *Staphylococcus caeli strain 82B (1)*  *Staphylococcus edaphicus CCM 8730* | | Air borne in rabbit farm  Air borne in rabbit farm  From sandy soil in Antartica |
| Bottle 5 | Y | | *Bacillus oleronius (0.88)*  *Bacillus oleronius (0.8429)*  *Bacillus oleronius (0.9000)* | *Heyndrickxia oleronia J19TS1 (0.997) Heyndrickxia oleronia J19TS1 (0.997)*  *Heyndrickxia oleronia J19TS1 (1)* | | High heat tolerance, from Dairy products  High heat tolerance, from Dairy products  High heat tolerance, from Dairy products |


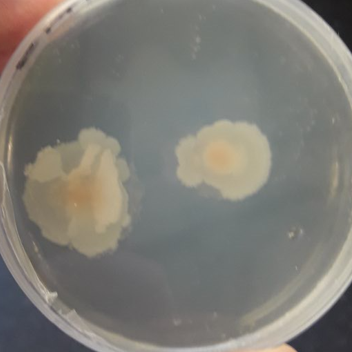

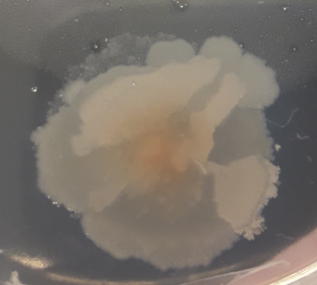

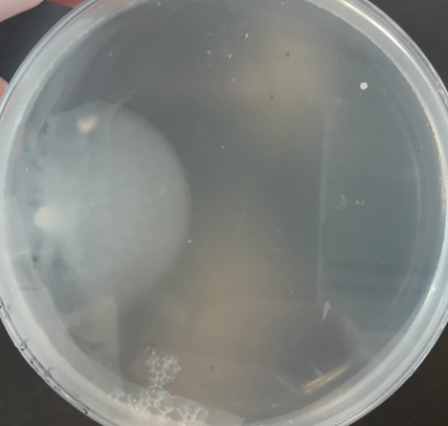

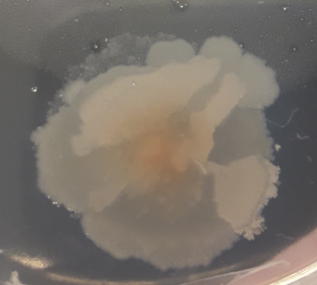


**A**

**B**

**Figure S2 A & B: Photographs of different colonies from the salt cavern brine growing on LB plates incubated at 30°C. The arrow shows a close-up of the colony on plate A.**

**Table S8: Identified microbial community with relative abundance in % (via 16S amplicon sequencing) from the growth enrichments. Confidence values based on RDP database are given in brackets.**

| **Additions** | **T [**°C] | **Community structure (confidence)** |
| --- | --- | --- |
| 10 mM glucose  0.2 % yeast extract  0.2 % peptone | 30 | *72% Halanaerobium* (1)  *28% Halanaerobacter* (0.99) |
|  | 80 | *43% Halanaerobium* (1)  *16% Halanaerobacter* (0.99)  5% Unknown Bacteria (1) |
| 20 mM lactate  20 mM acetate  0.04 % yeast extract | 30 | 48% Halanaerobiaceae (0.6)  25% *Halanaerobium* (1)  21% Desulfohalobiaceae (6 different ASVs with confidence between 0.6 – 0.85)  6% Unknown Bacteria (1) |
| 100 % H2  10 mM acetate  0.01 % yeast extract | 30 | 73% Halanaerobiaceae (0.6)  1.5% *Halanaerobium* (1)  24% Desulfohalobiaceae, 10 different ASVs (with confidence between 0.59 – 0.85)  0.7% Unknown Bacteria (1)  0.2% Nanohaloarchaeota (1) |

**Table S9: Measured H_2_S in ppm over time. Only bottles with H_2_S at some point are shown. All other bottles were at 0 ppm throughout the experiment**

| **T [°C]** | **Additions** | **Day 0** | **Day 94** | **Day 136** | **Day 176** | **Day 340** |
| --- | --- | --- | --- | --- | --- | --- |
| 30 | +ac +YE +H2 | 0 | 763 | 1450 | 780 | no sample |
| 30 | +ac +YE +H2 | 0 | 882 | 1561 | 977 | no sample |
| 30 | +YE | 0 | 425 | 983 |  | 1082 |
| 30 | +YE | 0 | 0 | 901 |  | 2084 |
| 30 | 100% H2 | 0 | 0 | 0 |  | 613 |
| 30 | 100% N2 | 0 | 0 | 37 |  | 0 |
| 80 | +ac +YE +H2 | 0 | 0 | 0 |  | 0 |
| 80 | +ac +YE +H2 | 0 | 0 | 0 |  | 401 |

**Table S10: Measured CH_4_ in ppm over time. Only bottles with CH_4_ at some point are shown. All other bottles were at 0 ppm throughout the experiment**

| **T [°C]** | **Additions** | **Day 0** | **Day 94** | **Day 136** | **Day 176** | **Day 340** |
| --- | --- | --- | --- | --- | --- | --- |
| 30 | +ac +YE +H2 | 0 | 0 | 0 | 1372 |  |
| 30 | +YE | 0 | 0 | 0 |  | 670 |

**Table S11: Measured Acetate in mM over time. Only bottles with significant changes in acetate are shown.**

| **T [°C]** | **Additions** | **Day 0** | **Day 100** | **Day 136** | **Day 170** | **340** |
| --- | --- | --- | --- | --- | --- | --- |
| 30 | +ac +YE +H2 | 20 | 20.3 | 19.2 | 17.2 |  |
| 30 | +ac +YE +H2 | 20 | 20.4 | 19.8 | 17.0 |  |
| 80 | +ac +YE +H2 | 20 | 20.8 |  | 20.7 | 22.3 |
| **T** | **Additions** | **Day 0** | **Day 56** |  |  |  |
| 30 | + glucose | 0 | 2.3 |  |  |  |

**Table S12: Measured pH over time.**

| **30°C** | **Day 0** | **Day 56** | **Day 94** | **Day 136** | **Day 176** | **Day 356** |
| --- | --- | --- | --- | --- | --- | --- |
| +ac +YE +H2 | 7.50 | 6.80 | 7.90 | 8.40 | 8.50 | 8.60 |
| +ac +YE +H2 | 7.50 | 7.20 | 7.90 | 8.40 | 8.40 | 8.10 |
| +H2/CO2 | 7.50 | 6.90 | 6.80 | 6.70 |  | 6.50 |
| +H2/CO2 | 7.20 | 6.90 | 6.70 | 6.70 |  | 6.50 |
| +YE | 7.20 | 7.20 | 7.40 | 7.60 |  | 7.10 |
| +YE | 7.30 | 7.30 | 7.20 | 7.40 |  | 7.20 |
| +H2/CO2 +vit | 7.20 | 6.70 | 6.60 | 6.60 |  | 6.55 |
| +H2/CO2 +vit | 7.20 | 6.80 | 6.70 | 6.80 |  | 6.50 |
| +form +H2/CO2 | 7.20 | 7.00 | 6.60 | 6.80 |  | 6.90 |
| +form +H2/CO2 | 7.20 | 7.00 | 6.60 | 6.70 |  | 6.80 |
| +100% H2 | 7.50 |  | 7.70 | 7.70 | 7.90 | 7.45 |
| +100% H2 | 7.50 |  | 7.70 | 7.75 | 7.70 | 7.45 |
| +100% N2 | 7.50 | 7.50 | 7.70 | 7.50 |  | 7.10 |
| autoclaved + H2 | 7.50 |  | 7.6 |  | 8 | 7.35 |
| autoclaved + H2 | 7.60 |  | 7.5 |  | 8 | 7.2 |
| **60°C** |  |  |  |  |  |  |
| +ac +YE +H2 | 7.5 | 7.6 | 7.5 | 7.5 |  | 6.6 |
| +ac +YE +H2 | 7.5 | 7.7 | 7.8 | 7.7 |  | 6.85 |
| +H2/CO2 | 6.9 | 6.5 | 6.5 | 6.3 |  | 6 |
| +H2/CO2 | 7 | 6.3 | 6.4 | 6.5 |  | 6.25 |
| +YE | 7.5 | 7.7 | 7.4 | 7.4 |  | 6.6 |
| +YE | 7.5 | 7.6 | 7.6 | 7.7 |  | 6.7 |
| +H2/CO2 +vit | 7 | 6.5 | 6.3 | 6.4 |  | 6 |
| +H2/CO2 +vit | 7 | 6.7 | 6.3 | 6.4 |  | 6.2 |
| +form +H2/CO2 | 7 | 6.6 | 6.4 | 6.4 |  | 6.1 |
| +form +H2/CO2 | 7 | 6.5 | 6.4 | 6.4 |  | 6.25 |
| +100% H2 | 7.5 |  | 7.3 | 7.2 |  | 6.55 |
| +100% H2 | 7.5 |  | 7.3 | 7.4 |  | 6.65 |
| +100% N2 | 7.5 | 7.3 | 7.3 | 7.4 |  | 6.5 |
| autoclaved + H2 | 7.50 |  | 7.5 |  | 6.9 | 6.25 |
| autoclaved + H2 | 7.50 |  | 7.4 |  | 6.4 | 6.7 |
| **80°C** |  |  |  |  |  |  |
| +ac +YE +H2 | 7.5 | 7.1 | 6.7 | 6.5 |  | 6.5 |
| +ac +YE +H2 | 7.5 | 7.1 | 6.8 | 6.8 |  | 6.3 |
| +H2/CO2 | 7.5 | 6.5 | 6.5 | 6.6 |  | 6.2 |
| +H2/CO2 |  | 6.4 | 6.5 | 6.6 |  | 6.2 |
| +YE |  | 7.6 | 7.3 | 7.2 |  | 6.6 |
| +YE |  | 7.5 | 7.2 | 6.9 |  | 6.3 |
| +H2/CO2 +vit |  | 6.4 | 6.5 | 6.4 |  | 6.3 |
| +H2/CO2 +vit |  | 6.4 | 6.5 | 6.6 |  | 6.3 |
| +form +H2/CO2 |  | 6.5 | 6.4 | 6.5 |  | 6.2 |
| +form +H2/CO2 | 7.4 | 6.5 | 6..5 | 6.6 |  | 6.3 |
| +100% H2 |  |  | 7.1 | 7 |  | 6.6 |
| +100% H2 |  |  | 7 | 7 |  | 6.5 |
| +100% N2 | 7.5 | 6.9 | 7.1 | 7 |  | 6.4 |
| autoclaved + H2 | 7.30 |  | 7.1 |  |  | 6.5 |
| autoclaved + H2 | 7.40 |  | 6.9 |  |  | 6.6 |

**Table S13: Gas analytics for the fermenting enrichments after 2 months of incubation**

|  | **Celsius** | **CO_2_ [%]** | **H_2_ [%]** |
| --- | --- | --- | --- |
| Untreated brine | 30 | 6.8 | 6.4 |
| Untreated brine | 30 | 3.2 | 1.7 |
| Untreated brine | 60 | 2.3 | 0.0 |
| Untreated brine | 60 | 0.8 | 0.0 |
| Untreated brine | 80 | 4.0 | traces |
| Untreated brine | 80 | 5.0 | 0.0 |
| Autoclaved brine | 30 | 5.8 | traces |
| Autoclaved brine | 60 | 0.8 | 0.0 |
| Autoclaved brine | 80 | 5.8 | traces |
